# Clinical Neuropathology practice guide 5-2015: MGMT methylation pyrosequencing in glioblastoma: unresolved issues and open questions 

**DOI:** 10.5414/NP300904

**Published:** 2015-08-07

**Authors:** Michal Bienkowski, Anna S. Berghoff, Christine Marosi, Adelheid Wöhrer, Harald Heinzl, Johannes A. Hainfellner, Matthias Preusser

**Affiliations:** 1 Institute of Neurology, Medical University of Vienna, Vienna, Austria,; 2 Department of Molecular Pathology and Neuropathology, Medical University of Lodz, Lodz, Poland,; 3 Department of Medicine I,; 4 Comprehensive Cancer Center-CNS Tumours Unit (CCC-CNS), and; 5 Center for Medical Statistics, Informatics, and Intelligent Systems, Medical University of Vienna, Vienna, Austria

**Keywords:** glioblastoma, management, prognosis, MGMT promoter methylation, pyrosequencing

## Abstract

O6-methylguanine-methyltransferase (*MGMT*) promoter methylation status has prognostic and, in the subpopulation of elderly patients, predictive value in newly diagnosed glioblastoma. Therefore, knowledge of the *MGMT* promoter methylation status is important for clinical decision-making. So far, *MGMT* testing has been limited by the lack of a robust test with sufficiently high analytical performance. Recently, one of several available pyrosequencing protocols has been shown to be an accurate and robust method for *MGMT* testing in an intra- and interlaboratory ring trial. However, some uncertainties remain with regard to methodological issues, cut-off definitions, and optimal use in the clinical setting. In this article, we highlight and discuss several of these open questions. The main unresolved issues are the definition of the most relevant CpG sites to analyze for clinical purposes and the determination of a cut-off value for dichotomization of quantitative *MGMT* pyrosequencing results into “*MGMT* methylated” and “*MGMT* unmethylated” patient subgroups as a basis for further treatment decisions.

## Introduction 

Glioblastoma is the most common primary brain tumor with inherently poor prognosis [1]. The current standard of treatment for glioblastoma patients aged 18 – 65 years is maximal safe resection of the tumor with subsequent radiotherapy and concomitant and adjuvant temozolomide-based alkylating chemotherapy (TMZ) [2]. Evidence from two randomized clinical trials supports stratification of therapy according to the methylation status of the O6-methylguanine-methyltransferase *MGMT* gene promoter in the population of elderly glioblastoma patients (> 65 years in the NOA-08 study and > 60 years in the Nordic Glioma study) [3, 4]. In these clinical trials, patients with an unmethylated *MGMT* promoter benefit more from radiotherapy alone, while patients with a methylated *MGMT* promoter benefit more from temozolomide chemotherapy alone. However, it must be noted that the interpretation of the true predictive value of the *MGMT* promoter methylation status has been limited in both trials, as the analytical performance of the assay was not investigated and *MGMT* test results were available in only a fraction of cases (56% in the NOA-08 study and 59% in the Nordic Glioma Study, respectively). In non-elderly glioblastoma patients, *MGMT* promoter methylation status does not fulfil the criteria of a predictive factor and does not directly guide therapeutic decisions, but it is a strong prognostic factor, which impacts clinical management [5]. For these reasons, *MGMT* testing is increasingly requested in the clinical setting and is recommended for routine clinical decision-making in elderly patients by current guidelines [6]. However, it remains unclear, which test method is most suitable for clinical *MGMT* testing, as several assays lack the necessary analytical performance (reproducibility and repeatability). 

Several recent studies show that *MGMT* pyrosequencing is a robust technique that offers valid, reliable and quick evaluation of the *MGMT* promoter methylation status from formalin-fixed and paraffin-embedded glioblastoma specimens and is therefore a rational candidate method for clinical purposes [7, 8, 9, 10, 11, 12, 13, 14, 15, 16, 17, 18, 19, 20, 21, 22, 23, 24, 25, 26, 27, 28, 29, 30, 31, 32, 33]. However, some uncertainties remain with regard to methodological issues, cut-off definitions, and optimal use of *MGMT* pyrosequencing in the clinical setting. This article aims at highlighting and discussing several of these open questions. 

## Methods 

### Methods of literature review 

The search for papers within the PubMed database was performed using the terms: “*MGMT* pyrosequencing” (76 results), “*MGMT* methylation glioma” (613 results), “*MGMT* methylation glioblastoma” (482 results), last performed on June 10, 2015. In this review we included only studies in which tissue samples of human glioblastoma were analyzed by *MGMT* pyrosequencing. 

### Open questions and unresolved issues 

**Which CpG sites should be analyzed?**

**Figure 1.**
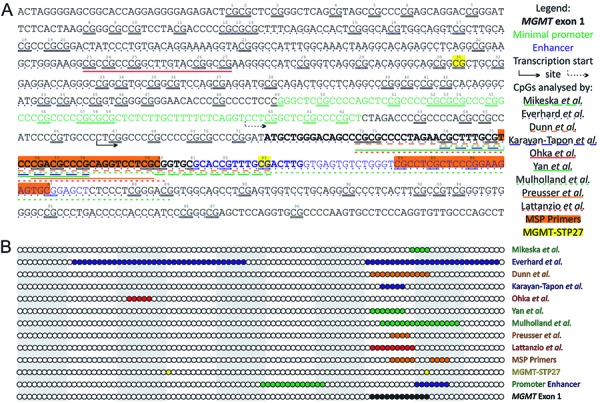
A: The annotated chromosome 10 sequence (positions: 129,466,653 – 129,467,461, gene build 19) with the *MGMT* CpG island (positions: 129,466,685 – 129,467,446) and short flanking sequences as well as a graphical legend. CpG sites within the island are marked with consecutive numbers and double underline. *MGMT* exon 1 is marked with bold. Minimal promoter and enhancer sequences (according to Harris et al. [35, 48]) are marked with green and blue, respectively. Transcription start sites are marked with solid arrow (according to gene build 19) and with dashed arrow (according to Harris et al. [48]). CpG sites analyzed in each publication are marked with a solid/dashed line underlining the sequence (the first author of the first paper analyzing given region is listed). MSP primers for the methylated sequence (according to Esteller et al. [49]) are marked with orange background. Two CpG sites included in the *MGMT*-STP27 model (based on HM-27K and HM-450K BeadChip arrays, according to Bady et al. [36]) are marked with yellow background. B: A simplified graphical representation of the *MGMT* CpG island with a circle representing each CpG site; alternating grey-white background marks decades of CpG sites. The sites belonging to each set are colored as above and explained in the legend on the right-hand side.

The *MGMT* promoter contains 98 individual CpG sites surrounding the transcription start site and comprises 2 differentially methylated regions (CpGs 25 – 50 and 73 – 90) (Figure 1) [34]. Due to the heterogeneous methylation of different CpG sites and the fact that most of the available methods for *MGMT* testing allow for the analysis of several sites, their proper selection seems crucial. Functional analyses showed that CpG 84 – 87 and 89 have the greatest impact on MGMT protein expression and co-localize with the minimal enhancer region (+143 to +201; containing binding sites for several transcription factors, including STAT3) [34, 35]. On the other hand, the analysis of CpGs 12 – 46 and 71 – 97 in glioblastoma patients showed that the methylation status of sites 27, 32, 73, 75, 79, 80 as well as means of 32 – 33 and 73 – 81 correlate best with the MGMT protein expression levels [9]. 

The methylation of the most commonly studied region, encompassing CpGs 74 – 78, was shown to significantly affect the prognosis for the patients with newly diagnosed glioblastoma [10, 12, 18, 19, 20, 22]. This region was described as relatively homogeneously methylated by Felsberg et al. [13], while as highly heterogeneous in some cases by Quillien et al. [20]. Nonetheless, the variation in the prognostic significance of different sites was relatively low [10, 12, 18, 20]. 

The mean methylation of the region containing CpGs 74 – 89 has shown significant correlation with survival of glioblastoma patients in three studies [21, 26, 27]. The observed methylation profile was often heterogeneous and, based on the optimal (outcome-wise) cut-off values, the closest correlation with overall survival (OS) and progression-free survival (PFS) was observed for CpGs 84, 89 as well as means 84 – 88, 74 – 78, and 76 – 80 according to the study by Quillien et al. [26], and for CpGs 85 and 87 according to the study by Collins et al. [27]. 

Additionally, the regions containing CpGs 23 – 27 [14, 15], 72 – 78 [17, 33], 72 – 80 [29], 72 – 83 [8, 28], 76 – 79 [25, 31], and 80 – 83 [7] were also analyzed, but the biological or clinical relevance of individual sites were not assessed. The prognostic significance was tested for CpGs 23 – 27 [14], 72 – 78 [33], 72 – 80 [29], and 72 – 83 [8, 28], and was proven for CpGs 72 – 80 [29], and 72 – 83 [8, 28]. The methylation of CpGs 23 – 27 and 80 – 83 was congruent with protein activity [15] and bisulfite sequencing [7], respectively. 

Finally, selecting more than one site for identification of the methylation status raises the issue whether all have the same biological or clinical relevance or not. The first scenario simply uses the mean of methylation levels for each site (as in most studies [8, 10, 11, 12, 13, 15, 16, 17, 19, 32, 32, 33]), the second one relies on a weighted approach (e.g., based on logistic regression as proposed for pyrosequencing by Mikeska et al. [7] or as MGMT-STP27, consisting of CpG 31 and 84, model for HM-450K and HM-27K BeadChip by Bady et al. [36]). 

In summary, there is a high heterogeneity among the studies in terms of the region analyzed, however, almost all of them focused on the region comprising exon 1 and enhancer (Figure 1). So far, best evidence exists for CpGs 74 – 78, as it is the best studied region, however, promising results have also been obtained with the analysis of larger regions (CpGs 72 – 83 and 74 – 89) and the distal part of the CGI (CpGs 84 – 89). Nonetheless, none of these analyses has been an adequately powered, prospective study. Thus, it remains unclear at the moment which region of the *MGMT* gene promoter is optimal for clinical decision-making. This question can only be resolved by further studies that aim at elaborating which CpG sites show the most relevant influence on patient outcome parameters. 

**Current use of *MGMT* status in clinical decision-making**

*MGMT* pyrosequencing yields a quantitative result, i.e., the percentage of methylated alleles for each of the investigated CpG sites. Some studies indicate that the continuous assay read-out carries prognostically relevant information, as higher methylation levels positively correlated with favourable patient survival times (Table 1). On note, application of a cut-off threshold to distinguish two patient groups (“*MGMT* methylated” and “*MGMT* unmethylated” patients) may lead to loss of clinically relevant information. As a consequence, in patients in whom treatment stratification is not dependent on *MGMT* promoter methylation status, i.e., the non-elderly patient population, continuous information on the percentage of methylated CpG sites in the *MGMT* promoter could be of interest for accurate prognostication of patient survival times. However, in patients in whom the *MGMT* status indeed is used to make treatment decisions, a distinct cut-off value is needed. At the moment this situation is given in the elderly population in whom the decision to recommend either radiotherapy (*MGMT* unmethylated or inconclusive test result) or chemotherapy (*MGMT* methylated) depends on the *MGMT* promoter methylation status. 

It is important to note, however, that data on the optimal predictive cut-off value to distinguish elderly glioblastoma patients likely to benefit from radiotherapy or chemotherapy are not available, so far. Such data would need to be generated from analyses of tissue specimens collected within adequately powered and randomized prospective clinical trials. In such trials, an interaction between therapy and *MGMT* promoter methylation status as assessed by pyrosequencing could be determined for overall survival. Of note, in the NOA-08 and the Nordic Glioma study, statistically significant interactions between therapy and *MGMT* status were not reported for patient overall survival [3, 4]. However, both studies used methylation-specific PCR for quantification of *MGMT* promoter methylation and the used cut-off is not transferable to pyrosequencing. The available studies applying *MGMT* pyrosequencing to glioblastoma patients were mostly retrospective in nature and included heterogeneously treated patients and were, thus, not adequately designed to determine *MGMT* methylation threshold levels for predictive purposes [7, 8, 9, 10, 11, 12, 13, 14, 15, 16, 17, 18, 19, 20, 21, 22, 23, 24, 25, 26, 27, 28, 29, 30, 31, 32, 33]. These studies used varying cut-off levels and some found a correlation of *MGMT* status with patient survival times with “*MGMT* methylated” patients generally having more favorable outcomes than “*MGMT* unmethylated” patients (Table 1). Although these studies suggest a prognostic role of *MGMT* promoter methylation status, they do not allow recommendations on a specific predictive cut-off value to distinguish “*MGMT* methylated” from “*MGMT* unmethylated” patients. 

In addition to *MGMT*, there are other (bio-)markers where optimal predictive cut-points would have to be determined – (biological) age, Karnofsky index/WHO performance status, and radiation dosage [3, 4]. Thereby mutual dependences between these cut-points could exist; e.g., for patients between 60 and 70 years another *MGMT* cut-point could be optimal to decide between radiotherapy and temozolomide chemotherapy than for patients > 70 years. Instead of hyping currently available cut-points and thereby pretending detailed knowledge, which actually does not exist yet, the definition of grey areas seems worth considering. They constitute biomarker intervals within which the decision for either of the two therapy options is appropriate; clear and well-founded therapy recommendations only exist for the extremes. 

**Studying MGMT is not over yet**

The published pyrosequencing protocols used for testing of *MGMT* promoter methylation status in glioblastoma are variable and differ especially with regard to the number and position of the tested CpG sites. A ring trial has shown high intra- and interlaboratory reproducibility for a commercially available *MGMT* pyrosequencing kit, whereas the analytical performance of other *MGMT* pyrosequencing protocols in glioblastoma is unclear [25]. 

*MGMT* promoter methylation status as determined by pyrosequencing seems to correlate with the survival prognosis of glioblastoma patients. However, so far available studies are limited by their retrospective nature, heterogeneity in patient populations, heterogeneity in pyrosequencing protocols (tested CpG sites), and the low patient numbers. Overall, *MGMT* promoter methylation status as determined by pyrosequencing seems to be a prognostic biomarker in glioblastoma patients, although the current level of scientific evidence is low. 

The predictive value of *MGMT* promoter methylation status as determined by pyrosequencing has not been addressed so far in systematic studies. The use of *MGMT* promoter methylation status as determined by pyrosequencing as predictive biomarker, e.g., for treatment decisions in elderly glioblastoma patients, is based on only weak and indirect scientific evidence, based on extrapolation of data elaborated with other methods of *MGMT* testing. 

For predictive biomarker studies the same evidence standards have to be applied as for adopting new therapies. Hence, these studies will usually be prospectively designed randomized controlled trials (RCTs). Note that for logistic or ethical reasons, in particular in rare diseases, the retrospective use of data from already performed RCTs would also be possible. Various specific prospective study designs for predictive biomarker studies have been suggested [37, 38, 39, 40, 41, 42, 43]; some with specific emphasis on determining predictive cut-points for continuously measured biomarkers [44, 45, 46]. 

Efficacy is the proof that a new treatment has sufficient therapeutic effect and is, among other things, necessary for marketing authorization decisions of regulatory bodies. Effectiveness is the treatment benefit in daily clinical routine when – among other things – no overly restrictive inclusion and exclusion criteria are in effect; it is assessed by comparative effectiveness research studies [47]. The scientific evaluation of predictive biomarkers like *MGMT* should focus on (1) efficacy, (2) effectiveness, and (3) the clarification of any major gaps between them; thereby (2) and (3) constitute key tasks of medical research in academic institutions. 

## Summary and conclusions

- *MGMT* pyrosequencing in glioblastoma has been shown to yield reproducible results within and between different laboratories
- *MGMT* pyrosequencing yields the percentage of methylated alleles for each of the investigated CpG sites as quantitative result
- The relative clinical relevance of the methylation status of individual CpG sites in the *MGMT* gene promoter is unclear and therefore open questions remain with regard to technical *MGMT* pyrosequencing assay setup, e.g., the selection of optimal PCR primers
- The available data from retrospective studies support a prognostic value of *MGMT* pyrosequencing results with higher percentages of methylated CpG sites in the *MGMT* gene promoter correlating with favorable survival times.
- A predictive cut-off value of *MGMT* pyrosequencing results for distinguishing *MGMT* methylated from *MGMT* unmethylated patients as a basis for treatment decisions has not yet been determined in adequately designed studies
- Further studies should aim to identify and validate an optimal *MGMT* pyrosequencing assay not only with high analytical performance, but also with proven clinical performance. Of particular relevance is the identification and validation of reasonable cut-off levels for the use as predictive biomarker, e.g., for treatment decisions in elderly glioblastoma patients.

## Conflict of interest 

The authors report no conflict of interest. 

## Acknowledgments 

M.B. is supported by the Healthy Ageing Research Centre project (REGPOT-2012-2013-1, 7FP). Special thanks to Prof. Thomas Ströbel, Institute of Neurology, Medical University of Vienna, for helpful comments. 

**Table 1.** Summary of all publications in which the *MGMT* promoter methylation was analyzed by pyrosequencing in human glioblastoma tissue. The data include: the number of glioblastoma samples analyzed by pyrosequencing and the fixation method, study type (whether prospective or retrospective and whether a clinical trial with homogeneous therapy or not), analyzed region (as the consecutive CpG numbers within the *MGMT* CpG island), applied threshold (all values listed if samples were divided into more than 2 groups; if different threshold values were used for the various sites, the range is shown in parentheses).

Samples	Material	Study type	CpGs	Threshold	Prognostic	Ref
22	Frozen	Retrospective	80 – 83	10%	N/A	[7]
109	Frozen, FFPE	Retrospective	72 – 83	9%, 29%	Yes	[8]
54	Frozen	Retrospective	12 – 46; 71 – 97	9%, 29%	N/A	[9]
81	Frozen	Retrospective	74 – 78	8%	Yes	[10]
17	FFPE	Retrospective	74 – 78	N/A	No	[11]
51	Frozen	Retrospective	74 – 78	10%, 27%	Yes	[12]
48	Frozen	Retrospective	74 – 78	8%	N/A	[13]
54	Frozen	Retrospective	23 – 27	14%	No	[14]
41	Frozen	Retrospective	23 – 27	N/A	N/A	[15]
15	FFPE	Retrospective	74 – 78	9%, 29%	N/A	[16]
77	Frozen	Retrospective	72 – 77	10%	N/A	[17]
41	FFPE	Prospective clinical trial	74 – 78	10% (11 – 45%)	Yes	[18]
86	Frozen	Retrospective	74 – 78	2.68%	Yes	[19]
100	Frozen	Retrospective	74 – 78	8%	Yes	[20]
182	Frozen	Retrospective	74 – 89	10%	Yes	[21]
166	Frozen	Retrospective	74 – 78	8%, 25%	Yes	[22]
78	FFPE	Retrospective	74 – 78	8%	Yes	[23]
64	Frozen, FFPE	Retrospective	74 – 78	5.72%, 20%, 35%	No	[24]
9	Frozen, FFPE, RCLPE	Retrospective	76 – 79	8%	N/A	[25]
89 + 50	Frozen	Retrospective	74 – 89	(4 – 32%)	Yes	[26]
225	FFPE	Prospective	74 – 89	10%	Yes	[27]
128	Frozen	Retrospective	72 – 83	10%	Yes	[28]
46	Frozen, FFPE	Retrospective	72 – 80	9%	Yes	[29]
43	FFPE	Retrospective	74 – 89	10%	N/A	[30]
99	FFPE	Retrospective	76 – 79	8%	N/A	[31]
303	FFPE	Retrospective	74 – 78	9%	Yes	[32]
105	Frozen	Retrospective	72 – 77	10%	No	[33]
